# Female novelty and male status dynamically modulate ejaculate expenditure and seminal fluid proteome over successive matings in red junglefowl

**DOI:** 10.1038/s41598-019-41336-5

**Published:** 2019-04-10

**Authors:** Aitor Alvarez-Fernandez, Kirill Borziak, Grant C. McDonald, Steve Dorus, Tommaso Pizzari

**Affiliations:** 1 Edward Grey Institute, Department of Zoology, University of Oxford, Oxford, OX1 3SZ UK; 2 Center for Reproductive Evolution, Syracuse University, 107 College Place, Syracuse, NY 13244 USA

## Abstract

Theory predicts that males will strategically invest in ejaculates according to the value of mating opportunities. While strategic sperm allocation has been studied extensively, little is known about concomitant changes in seminal fluid (SF) and its molecular composition, despite increasing evidence that SF proteins (SFPs) are fundamental in fertility and sperm competition. Here, we show that in male red junglefowl, *Gallus gallus*, along with changes in sperm numbers and SF investment, SF composition changed dynamically over successive matings with a first female, immediately followed by mating with a second, sexually novel female. The SF proteome exhibited a pattern of both protein depletion and enrichment over successive matings, including progressive increases in immunity and plasma proteins. Ejaculates allocated to the second female had distinct proteomic profiles, where depletion of many SFPs was compensated by increased investment in others. This response was partly modulated by male social status: when mating with the second, novel female, subdominants (but not dominants) preferentially invested in SFPs associated with sperm composition, which may reflect status-specific differences in mating rates, sperm maturation and sperm competition. Global proteomic SF analysis thus reveals that successive matings trigger rapid, dynamic SFP changes driven by a combination of depletion and strategic allocation.

## Introduction

Sperm competition, arising from polyandry, favours the production of competitive ejaculates^1–3^. Producing competitive ejaculates however, is costly and males are expected to maximize their reproductive success by strategically tailoring ejaculate expenditure to the value of a mating event^4,5^. A number of factors are predicted to dictate patterns of male ejaculate expenditure, including the level of sperm competition likely experienced by an ejaculate and female phenotypic traits^4,5^. For example, a number of studies have demonstrated male preferential investment in certain female phenotypes linked to reproductive investment through simultaneous binary choice experiments^6,7^. While these experiments have proved helpful in elucidating fundamental aspects of ejaculate expenditure decisions, they present males with a rather unrealistic choice between two simultaneously available females. In a more realistic situation relevant to many polygynandrous mating systems, a male encounters females of variable reproductive quality sequentially^8^. This presents males with a sequential decision-making task in which investment in the current mating opportunity curtails allocation to any sexually novel female that they may encounter in the near future (i.e. before amale’s sperm and seminal fluid (SF) reserves can be fully replenished, which can take several days, e.g.^6^). A simple way through which males can optimise performance in this task, is if recent expenditure in the current female inhibited further investment in the same female, thus saving resources for a new female. Males are therefore expected to modulate ejaculate expenditure in response to the sexual novelty of a female; reducing investment with increasing female sexual familiarity, and preferentially investing in sexually novel females^5,8,9^.

Consistent with these expectations, males of a range of polygynandrous species have been shown to prefer sexually novel partners, a behavioural response known as the Coolidge effect. The Coolidge effect refers to a progressive decline in a male’s sexual interest in a female as the male becomes progressively more sexually familiar with this female (i.e. through successive matings) and the subsequent resumption of his mating propensity on encountering a sexually novel female^9–12^. Increasing evidence indicates that – in addition to differential male mating propensity- some males may also respond to female sexual novelty through a pattern of ejaculate expenditure that recapitulates the Coolidge effect^13–15^. For example, when male feral domestic fowl *Gallus domesticus*, and red junglefowl, *G. gallus* were allowed to copulate *ad libitum* with a sequence of females, average ejaculate size (i.e. the number of sperm contained in an ejaculate) displayed a sharp overall decline over successive copulations as a result of depletion of a male’s extragonadal sperm reserves. However, ejaculates delivered to sexually novel females on average contained a higher number of sperm than one would predict based on the overall pattern of sperm depletion, indicating that males progressively reduce ejaculate expenditure with increasing sexual familiarity and bias sperm allocation in favour of sexually novel females^6^. All else being equal, the extent to which a male should withdraw his ejaculate expenditure in the current female should reflect his likelihood of encountering a new female in the near future. This likely explains why the Coolidge effect is not observed in monogamous species of rodents^9^ and crickets^16,17^, in which males do not normally encounter or mate with multiple females, and may also explain variation across individuals within populations. For example, in the externally fertilizing European bitterling *Rhodeus amarus*, females place their eggs in the gills of freshwater mussels through their exhalant siphon, and males fertilize eggs by releasing sperm over the inhalant siphon of the mussel. Each new mussel therefore, represents a novel fertilization opportunity for a male. Socially dominant males, which aggressively defend territories to monopolize access to both mussels and females, increase the number of ejaculations (a proxy of sperm investment), on encountering a new mussel compared with a familiar one^18^. Socially subdominant males on the other hand, tend to allocate most sperm to the first mussel encountered regardless of whether this is novel or familiar^18^. It is likely that these status-specific responses reflect differences in the availability of fertilization opportunities.

Most studies of strategic ejaculate expenditure by males have focused on sperm allocation^4,5,7^, ignoring the potential role of seminal fluid. Seminal fluid, the physiological, non-cellular component of an ejaculate, is a complex medium characterised by a multitude of molecules, including peptides and proteins. Several of these have been shown to play a key role at different stages of the reproductive event, e.g. by modulating sperm fertilising efficiency and longevity, antimicrobial defences, female sperm utilisation, the coordination of ovulation and oviposition in females, maternal allocation, and female remating propensity^19–24^. Given their roles, males might also be expected to strategically invest in certain SF proteins (SFPs). Moreover, theoretical work has shown that explicit consideration of potential SF effects can drastically change predictions of evolutionarily stable strategies of male ejaculate expenditure, calling for empirical research to elucidate patterns of male SF allocation^25–27^. Consistent with this expectation, empirical evidence is beginning to emerge suggesting that patterns of SFP allocation might vary with the level of sperm competition^28–33^ and access to new mating opportunities as determined by male social status^34^. Most of this recent work however, has relied on gene expression levels or protein composition in male tissues. While informative, these approaches do not capture rapid dynamic changes in SFP allocation across individual ejaculates, making it difficult to attribute changes in SFP levels to differential SFP production *versus* differential allocation to individual mating opportunities.

Here, we study patterns of variation in sperm and SF investment in relation to female sexual novelty in male red junglefowl. Red junglefowl and natural populations of its domestic descendent, the domestic fowl have a well characterised polygynandrous mating system in which sperm competition is typically intense^35^. Socially dominant males often have access to more females^36^ and on average face a lower level of sperm competition than subdominant males^36,37^. Male fowl gain reproductive success by mating repeatedly with the same female^36,37^, however their extragonadal sperm reserves deplete rapidly over successive matings^6^, creating scope for strategies of preferential ejaculate expenditure. Consistent with this, male fowl have been shown to alter sperm allocation in response to socio-sexual conditions, including the sexual familiarity of a female^6^. SF allocation to ejaculates may also vary dynamically and changes in SF composition have been evoked to explain intra-male variation in sperm swimming velocity^38^, including variation associated with changes in social status^39^. The recent characterisation of the red junglefowl seminal fluid proteome identified more than 1,100 SFPs including some potentially involved in immunity and antimicrobial defences, sperm maturation, and fertilisation, revealing that this is a functionally complex medium with potentially important effects in sperm competition and postcopulatory events^40^.

We used an experimental protocol in which each male was exposed to, and allowed to mate up to 5 consecutive times with a first female, before immediately being exposed to a sexually novel, second female and allowed to mate one final time with this female. We first describe patterns of variation in ejaculate expenditure in terms of sperm numbers and SF volume over the mating sequence. We then use high-throughput mass spectrometry to characterise dynamic patterns of SF proteome variation in the subset of dominant and subdominant males that produced adequate ejaculates with sufficient amounts of seminal fluid for proteomic analysis, across three matings with the first female and in the single mating with the second, novel female.

## Results

### Ejaculate expenditure

**Figure 1:**
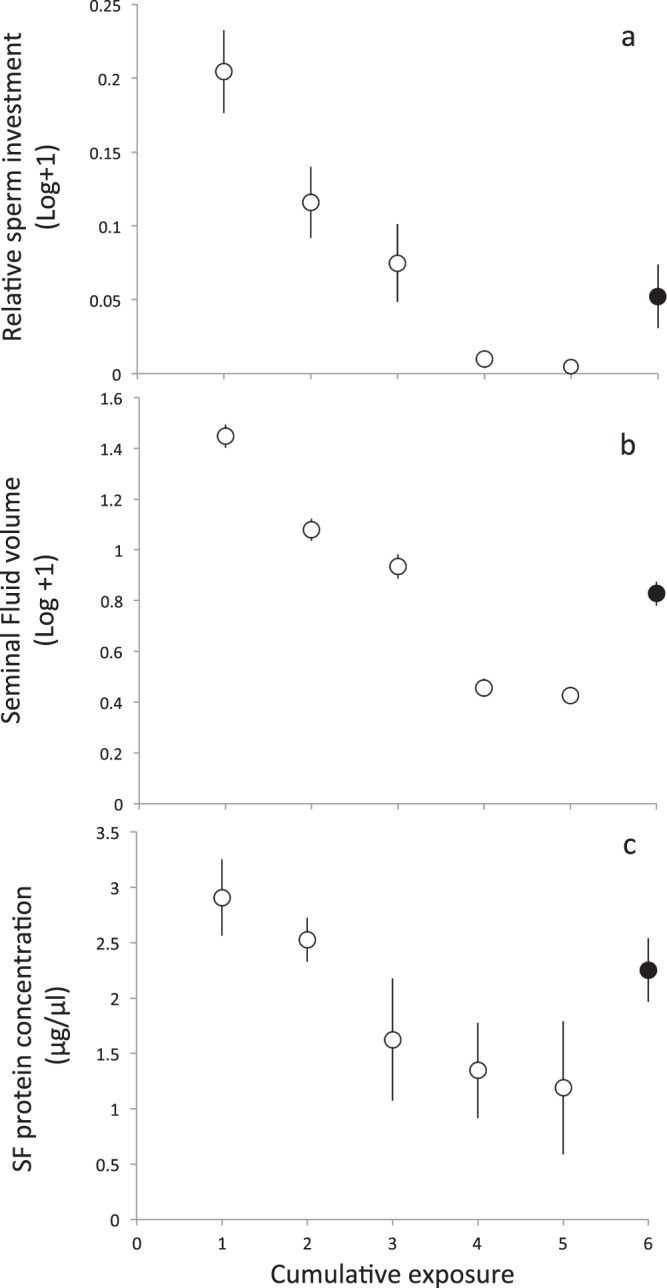
Patterns of ejaculate investment over a mating sequence. (**a**) Average number of sperm (Log-transformed, standardised against maximum number of sperm for each male) over the mating sequence comprising the first five matings with the first female (empty data points), and the 6^th^ mating with a novel, second female (filled data point). (**b**) The average volume of seminal fluid (SF) allocated by males across the six mating opportunities. (**c**) The average proportion of SF protein present in the ejaculates produced over the mating sequence. Vertical bars represent SEM. Note that (**a**) and (**b**) include mating opportunities that resulted in no ejaculation, thus capturing both male mating propensity and ejaculate investment when mating occurred (see Figs S1–S3 for additional information).

Variation in average sperm numbers over the mating sequence followed the typical pattern previously described for this species^6^, declining steadily over successive copulations with the first female and increasing to some degree in the first mating with the second, sexually novel female (Fig. 1a). We analysed this pattern in two ways: first, variation in the probability of a male producing sperm (probability of ejaculation) at a given mating opportunity in a trial, and second, variation in the number of sperm contained in an ejaculate in cases where males did produce sperm. Variation in the average probability of ejaculation was explained by cumulative exposure (i.e. successive matings) and female novelty (i.e. the first female with whom the male becomes progressively sexually familiar over successive matings *vs* the second sexually novel female; see methods). Both the model with the lowest AIC and the most parsimonious model retained both cumulative exposure and female novelty as predictors (Table 1). Estimates based on model averaging indicated that, as expected, cumulative exposure had a negative effect on the probability of producing sperm at a given mating opportunity, consistent with the effect of sperm depletion, while female novelty had a positive effect, increasing the probability of producing sperm for a given amount of exposure, consistent with preferential male investment in sexual novelty (Supplementary Table S1). The most parsimonious models for sperm numbers retained either only cumulative exposure or cumulative exposure and female novelty (Table 2). Model averaging of these models indicated that cumulative exposure had a negative impact on sperm numbers, while female novelty had a positive effect, confirming sperm depletion and preferential investment in novel females (Supplementary Table S2). The Wilcoxon rank sum test confirmed this, indicating a tendency for the first copulation with the second novel female to deliver more sperm than one would predict based on the pattern of sperm decline observed in a male over his previous matings with the first familiar female (V = 67, p = 0.031). This overall response however masked substantial individual differences (Supplementary Figs S1a and S2).

**Table 1:** Effect of Cumulative Exposure (CE) and Female order (F) on probability of ejaculation.

No.	Fixed effects	*k*	Log-likelihood	*AIC*_*c*_	Δ*AIC*_*c*_	Weight
1	CE + F + CE × F	5	−50.81	112.34	0.00	0.57
**2**	**CE + F**	**4**	−**52.65**	**113.77**	**1.43**	**0.28**
3	CE	3	−54.42	115.11	2.77	0.14
4	*Null*	2	−59.16	122.46	10.13	0.00
5	F	3	−58.84	123.95	11.62	0.00

All models include a random effect for male identity. Fixed effects: CE = Cumulative Exposure, F = Female order. Models are ranked by increasing *AIC*_*c*_ values. For each model we show: fixed effects, number of estimated parameters (*k*), log-likelihood, *AIC*_*c*_, Δ*AIC*_*c*_ (difference between given model and model 1) and the Akaike weight. The most parsimonious models are above the dashed line, and the best minimal model is in bold. Models included 90 observations from 19 males.

**Table 2:** Effect of Cumulative Exposure (CE) and Female order (F) on variation in the number of sperm ejaculated.

No.	Fixed effects	*k*	Log-likelihood	*AIC*_*c*_	Δ*AIC*_*c*_	Weight
1	CE + F	5	−82.33	175.88	0.00	0.48
**2**	**CE**	**4**	−**83.74**	**176.28**	**0.40**	**0.39**
3	CE + F + CE × F	6	−82.32	178.39	2.51	0.14
4	F	4	−91.82	192.43	16.55	0.00
5	*Null*	3	−93.19	192.84	16.96	0.00

All models include a random effect for male identity. Fixed effects: CE = Cumulative Exposure, F = Female order. Models are ranked by increasing *AIC*_*c*_ values. For each model we show: fixed effects, number of estimated parameters (*k*), log-likelihood, *AIC*_*c*_, Δ*AIC*_*c*_ (difference between given model and model 1) and the Akaike weight. The most parsimonious models are above the dashed line, and the best minimal model is in bold. Models included 55 observations from 18 males.

Patterns of variation in average SF volume largely mirrored patterns of sperm numbers (Fig. 1b), indicating that average volume declined over successive matings with the same female and increased to some degree in the presence of a second, sexually novel female. Two of the three most parsimonious models retained both cumulative exposure and female novelty, while one retained only cumulative exposure (Table 3; Supplementary Table S3). The Wilcoxon rank sum test confirmed this result, indicating that the first copulation with the second novel female delivered significantly more seminal fluid than expected based on the pattern of decline in SF volume experienced by a male over his matings with the first familiar female (V = 70, p = 0.017; Supplementary Figs S1b and S3).

**Table 3:** Effect of Cumulative Exposure (CE) and Female order (F) on variation of SF volume.

No.	Fixed effects	*k*	Log-likelihood	*AIC*_*c*_	Δ*AIC*_*c*_	Weight
**1**	**CE**	**4**	−**75.37**	**159.58**	**0.00**	**0.38**
2	CE + F	5	−74.47	160.21	0.63	0.27
3	CE + F + CE × F	6	−73.50	160.83	1.25	0.20
4	*Null*	3	−77.81	162.12	2.54	0.11
5	F	4	−77.55	163.94	4.36	0.04

All models include a random effect for male identity. Fixed effects: CE = Cumulative Exposure, F = Female order. Models are ranked by increasing *AIC*_*c*_ values. For each model we show: fixed effects, number of estimated parameters (*k*), log-likelihood, *AIC*_*c*_, Δ*AIC*_*c*_ (difference between given model and model 1) and the Akaike weight. The most parsimonious models are above the dashed line, and the best minimal model is in bold. Models included 53 observations from 17 males.

Variation in the SF protein concentration displayed a similar -albeit less pronounced- pattern, indicating that the protein content of the SF declined over cumulative exposure through depletion, and suggested that it might increase in some males in response to female novelty (Fig. 1c; Table 4; Supplementary Table S4). Inconsistent with the latter suggestion however, the Wilcoxon rank sum test did not detect a significant overall difference between the SF protein content of the ejaculate that a male produced with the second novel female, and expectations of his allocation to this female based on patterns of variation in his ejaculates with the first familiar female (V = 22, p = 0.219; Supplementary Figs S1c and S4).

**Table 4:** Effect of Cumulative Exposure (CE) and Female order (F) on SF protein concentration.

No.	Fixed effects	*k*	Log-likelihood	*AIC*_*c*_	Δ*AIC*_*c*_	Weight
**1**	**CE**	**4**	−**32.16**	**73.74**	**0.00**	**0.48**
2	CE + F	5	−31.62	75.46	1.72	0.20
3	CE + F + CE × F	6	−30.26	75.75	2.01	0.17
4	*Null*	3	−35.04	76.91	3.17	0.10
5	F	4	−34.45	78.33	4.59	0.05

All models have the random effect for male identity. Fixed effects: CE = Cumulative Exposure, F = Female order. Models are ranked by increasing *AIC*_*c*_ values. For each model we show: fixed effects, number of estimated parameters (*k*), log-likelihood, *AIC*_*c*_, Δ*AIC*_*c*_ (difference between given model and model 1), and the Akaike weight. The most parsimonious models are above the dashed line, and the best minimal model is in bold. Models included 33 observations from 7 males.

Overall, we found no indication that male status affected patterns of ejaculate expenditure. Compared to subdominants, socially dominant males invested proportionally more sperm and SF in their ejaculate with the second female than in their last ejaculate with the first female; and subdominant males invested proportionally more SF protein in the second female than dominants; however, these differences were not statistically significant (Supplementary Tables S5 and S6).

### Seminal fluid proteome variation

To achieve a refined and complementary molecular perspective on SF variation, high throughput tandem mass spectrometry (MS/MS) was used to characterize proteome composition of SF for both dominant and subdominant males across the first three matings with the first female and the single mating with the second, novel female. A total of 1685 SFPs were robustly identified by 113,365 peptide spectral matches (Supplementary Table S7). The number of SFPs identified per mating ranged from 1091 to 1305, with 805 proteins identified consistently across all four successive matings.

**Figure 2:**
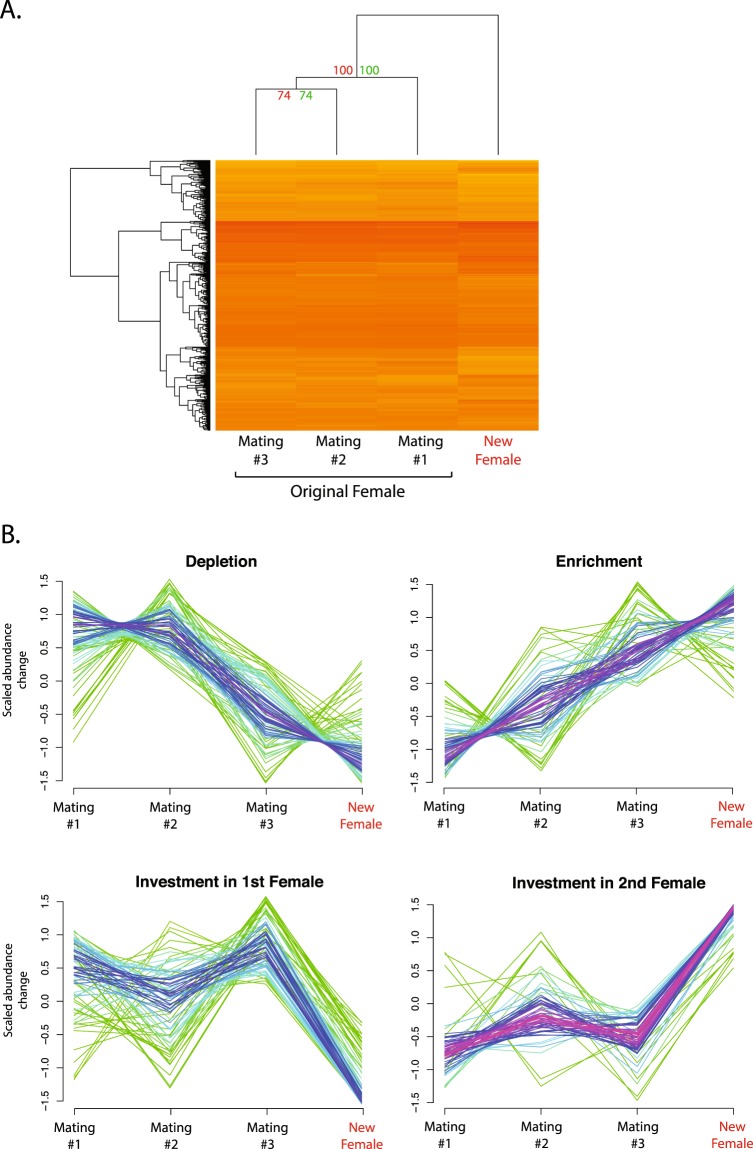
Seminal fluid proteome dynamics across successive matings. (**A**) Hierarchical clustering analysis. SF samples across four successive matings. Proteins were organized by dendrogram order, with the distance matrix computed using the euclidean method. AU (Approximately Unbiased) (red) *p*-value and BP (Bootstrap Probability) (green) values were computed by multiscale bootstrap resampling. (**B**) Fuzzy clustering analysis was conducted to identify global patterns of protein abundance differences that are associated with the mating sequence. Protein abundance patterns relative to the cluster average are depicted for each protein (purple: high identity; green: lower identity). Note: For simplicity, only one of two clusters exhibiting a pattern of protein depletion is displayed.

We first characterized patterns of molecular variation in SF composition without consideration of male social status. We note that our analysis involved pooling equal amounts of SF protein across two dominant and two subdominant males, respectively, and standardizing the quantity of protein analysed by MS/MS across samples. We therefore analysed compositional dynamics of SF proteomes and discuss how this relates to absolute abundance changes where possible. Hierarchical clustering analysis with bootstrapping revealed statistically robust distinctions between SF proteomes associated with the first female (all three matings) and the novel, second female (Fig. 2A; bootstrap prob. = 100). Interestingly, the SF proteome in the first mating could also be distinguished from the subsequent two matings with the first female (bootstrap prob. = 74). In fact, the greatest similarity was observed between mating two and three with the first female (R-square = 0.93), while the greatest variation was observed between the first mating with the first female and the mating with the second female (R-square = 0.60). Principal Component Analysis (PCA) results were consistent with the hierarchical clustering analysis and captured interproteomic variation that distinguished the first mating with the first female from the mating with the second female (Supplementary Fig. S5).

To explore emergent patterns and potential functional significance of SFP variation, a fuzzy-clustering analysis was implemented (Fig. 2B). First, 43% of proteins decreased in abundance across sequential matings, consistent with protein depletion. It is noteworthy that total SF protein quantity was comparable or decreased in these males, therefore these compositional decreases also reflect absolute decreases (i.e. depletion). As might be expected given the large number of depleted proteins, limited functional coherence was observed. Second, a smaller subset of proteins (19%) exhibited progressive abundance increases over the mating sequence. The vast majority of these increases reflect absolute abundance increases across matings with the first female and compositional increases in the mating with the novel female given the smaller amount of total SF protein present. This cluster contained a 1.4-fold excess of plasma proteins (p = 0.028), including large abundance increases amongst many of the most abundant SFPs (e.g. serum albumin precursor) and major plasma components (e.g. ovotransferrin, apolipoprotien A-1 and hemoplexin)^40^. Additionally, this cluster contained a highly significant 2.8-fold enrichment of immunity proteins (p < 0.0001), including proteins involved in the regulation of complement activation (p = 0.034). Diverse complement immunity proteins are present in sperm in mammals and have been hypothesized to regulate complement attack in the female reproductive tract^41^. Consistent with the widespread presence of proteolytic pathways in SF^40^, this cluster also exhibited a highly significant enrichment of negative regulators of endopeptidase activity (p < 0.0001). Lastly, two clusters of SFPs were identified where protein abundance increases (21%) or decreases (17%) were specific to the mating with the second, novel female. Amongst those proteins that increased in compositional abundance when mating with the novel female, we observed a significant enrichment of categories relevant to sperm composition and function, including microtubule motor activity (p = 0.0004), inner dynein arm assembly (p = 0.003), and cilium movement (p < 0.0001). This is consistent with the significant excess of sperm proteins membership observed within this cluster (7.8-fold; p < 0.0001). We next examined the distribution of membership probability per cluster, which reflects the level of similarity in the pattern of abundance for each protein relative to the cluster average. This revealed that proteins preferentially invested in the second female had significantly higher membership values than all other clusters (all p < 0.001; Fig. S6). Additionally, proteins with enriched patterns of abundance had significantly higher membership values than the cluster containing depleted proteins or those preferentially invested in the first female (all p < 0.05). Thus, compositional protein abundance increases associated with the second, sexually novel female exhibit patterns of tight co-expression.

**Figure 3:**
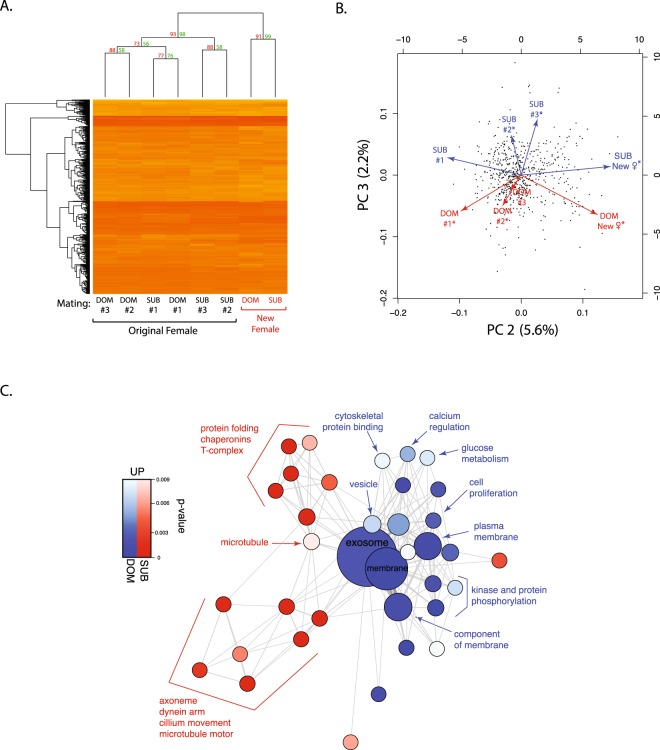
Seminal fluid proteome dynamics in dominant and subdominant males. (**A**) Hierarchical clustering analysis. SF samples across four successive and proteins were organized by dendrogram order, with the distance matrix computed using the euclidean method. Approximately Unbiased *p*-value (red) and Bootstrap probability (green) value were computed by multiscale bootstrap resampling. (**B**) Principal component analysis of SF samples from dominant and subdominant males across four successive matings. Principal components 2 and 3 are displayed. (**C**) Piano analysis displays enriched functional groups of proteins that differ in abundance between the SF sample of dominant and subdominant males when mated to the second, novel female. Node size is proportional to the total number of proteins in each functional category (total number of proteins in the comparison = 746).

We next assessed the contribution of social status to these patterns. Consistent with our previous hierarchical clustering analysis, there was a robust distinction between SF proteomes, for both dominant and subdominant males, associated with the first female and those with the second, novel female (Fig. 3A, bootstrap prob. = 98). It is also noteworthy that the first mating with the first female and the mating with the second female resulted in proteomes with similar characteristics from males of both social status, respectively. For example, despite the fact that all SF proteomes were significantly correlated (average R-square = 0.78), the highest correlation was observed between dominant and subdominant males mating with the first female (R-square = 0.90). The SF proteomes produced by males of each social status during the mating with the second female were also highly correlated (R-square = 0.88). Further analysis of SFP abundance using PCA identified 8 PCs, including 5 significant PCs (Supplementary Table S7), accounting for all of the variation. PC1 (86.4% of the variance) captured variation in SFP abundance (see Methods). PC2 (5.6%) captured interproteomic variance across the mating sequence, including significant negative correlations between PC2 loadings and SFP abundance for the first mating with the first female (dominant p < 0.001; subdominant p < 0.001), positive correlations for the mating with the second female (dominant p < 0.001; subdominant p < 0.001) and a marginally negative correlation for the second dominant mating with the first female (p = 0.044). PC3 (2.2%), however, consistently distinguished between the proteomic profiles of dominant and subdominant males (Fig. 3B). This included significant positive correlations for the first two dominant male matings with the first female (all p < 0.001) and the second and third subdominant mating with the first female (all p < 0.0001). PC4–8 captured a combined total of 4.3% of the variance and did not include consistent relationships with the mating sequence or male status.

We investigated the basis of variation between dominant and subdominant males captured by PC3 by directly comparing protein abundance between dominant and subdominant samples for the mating with the sexually novel, second female (Fig. 3C). The SF samples of subdominant males exhibited an enrichment of proteins with sperm flagellar functions, such as axoneme, inner dynein arm, microtubule motor, cilium movement and sperm motility. In contrast, the SF samples of dominant males showed increased abundance of exosome proteins (p = 9.60^−6^), a class of proteins that we have previously suggested contributes to post-testicular sperm maturation in this species^40^. This pattern included significant abundance differences in annexin A1 and A7 and enriched directional changes in other exosomal marker proteins, such as 14-3-3 eta, Rab-1A and Rab-14, as well as vesicle and cell proliferation-regulating proteins. Other Rab family members were associated in our previous study with increased sperm quality in older males^40^.

## Discussion

Patterns of male ejaculate expenditure have been the focus of intense research interest^4,5,7^. However, few studies have: (**a**) investigated male responses to a temporal sequence of mating opportunities, a scenario typical of most polygynandrous species; (**b**) considered male investment in SFPs in addition to sperm; and (**c**) implemented molecular analyses to characterize the specific proteomic basis of male investment. In this study, we sought to address these important gaps in our knowledge.

Because the mating system of most organisms is characterised by some degree of polyandry and polygyny, a large component of a male’s fitness is determined by the way a male balances ejaculate expenditure to defend his paternity of the ova of a particular female and to fertilise the ova of other females. The interaction between sperm competition and sperm depletion is likely to shape patterns of ejaculate variation in many species exhibiting some degree of polyandry and polygyny. Typically, a male will encounter mating opportunities sequentially, and strategies of ejaculate expenditure should seek to optimise investment in a current female given a certain probability of encountering a new female in the near future. Preferential investment in sexually novel females along a sequence of mating events would represent a pattern of ejaculate expenditure consistent with the Coolidge effect. The present results indicate that on average, male red junglefowl tend to reduce sperm allocation over successive copulations with the same female and increase it upon exposure to a new, sexually novel female, confirming previously described male preferential sperm investment in sexually novel females in this^6^ and other polygynandrous species^13–15,18,42^. These findings are also consistent with previous studies demonstrating a more general male preference for sexually novel partners^9,12,43,44^.

Our results additionally reveal that changes in SF volume follow a similar pattern of depletion and preferential investment in novelty, and that for a given volume of seminal fluid, ejaculates further down the mating sequence tend to contain a lower amount of SFPs, indicating that investment in SF protein concentration is particularly sensitive to depletion. There was also some suggestion that males might preferentially increase SF protein concentration when mating with the second, sexually novel female, however this effect was weak at best, given high inter-male variation. One possible factor contributing to inter-male variation may be that individual males were each exposed to a single experimental trial, which prevented within-male replication and estimates of individual variation. Absence of replication also prevented males from adjusting to the regime of female availability imposed by the experiment, e.g. by learning to expect a second female. While we know very little about the role that cognition plays in the development of male ejaculate expenditure strategies^5^, repetition of the experimental protocol should promote a male’s investment in sexual novelty by reinforcing expectation of a second female in a trial. Contrary to our expectations, we found no indication that social status influenced ejaculate expenditure, although we cannot rule out that inter-individual variation unrelated to male status may have limited the power of our analyses to test for status effects.

Importantly, we characterized SFP variation over the mating sequence using a high-throughput proteomic approach. To the best of our knowledge, this is the first proteomic study to investigate SF composition across a mating sequence and between males of different social status. We have previously shown that the seminal fluid proteome of male red junglefowl is complex, consisting of over 1,000 SFPs, some of which have putative roles in anti-microbial responses, innate and adaptive immunity, post-spermatogenic sperm maturation, reproductive ageing and sperm velocity^40^. Part of this complexity stems from the fact that two distinct secretions contribute to the SF of natural ejaculates; the seminal plasma produced by the testis and vas deferens, and the transparent fluid, produced by the lymphatic folds of the cloaca at ejaculation^45^, which may be a major contributor of plasma proteins such as serum albumin, transthyretin and ovotransferrin^40^.

The results of our study confirm the proteomic complexity of red junglefowl SF, and –critically– reveal that the SF proteome undergoes marked changes over the mating sequence. Hierarchical clustering and principal component analyses demonstrate that a large proportion of SFP variation is explained by the fact that the ejaculates allocated to the second, novel female were markedly different from the proteomic profiles of the seminal fluid of ejaculates produced with the first female. If sexual novelty were the sole determinant of the SF proteome, we would expect the profile of the first ejaculate with the first female to be similar to the first ejaculate with the second female. Conversely, if depletion were the sole determinant of SF proteome, we would expect the profile of the first mating with the second female to be similar to that of the last mating with the first female. However, both statistical analyses show that the proteomic profiles of ejaculates allocated to the second, sexually novel female had a distinct signature compared to the seminal fluid allocated to the first three consecutive ejaculates with the first female. We therefore conclude that a combination of depletion and preferential investment in sexual novelty contribute to the unique composition of SFPs allocated to the second female.

Physiological constraints on SFP production and investment are supported by the identification of a large group (43%) of SFPs that exhibit a gradual depletion over the mating sequence. Thus, depletion is the predominant source of variation across the mating sequence. The identification of a second group of SFPs that increase in abundance suggests that these proteins might compensate for depletion of other SFPs. These proteins are highly enriched for plasma proteins, which tend to be amongst the most abundant proteins in the SF proteome, suggesting that lymphatic folds of the cloaca may be the predominant contributor to SF across successive matings. Although it would be premature to draw specific conclusions, these results inform non-mutually exclusive functional hypotheses. First, a parsimonious explanation for the observed enrichment of plasma proteins is that these proteins might be cheaper to produce than seminal plasma SFPs and might function as ‘cheap fillers’. If female postmating response, e.g. in terms of propensity to mate again with other males, is modulated by the volume of semen received, depleted males might be able to discourage female remating by preferentially investing in lymphatic fold plasma secretions (i.e. the transparent fluid). A similar mechanism has been suggested for parasperm in green-veined butterflies *Pieris napi*. In this species, males produce two distinct categories of sperm cells, eusperm, which fertilise the ova, and parasperm, which are more motile but do not fertilise. The significance of parasperm remains an evolutionary paradox. However, males that inseminate a larger proportion of parasperm are able to delay female remating, thus providing their eusperm with a fertilising advantage^46^. For both, plasma SFPs and parasperm, the assumption is that these are cheaper to produce than other SFPs and eusperm, respectively. Second, over successive matings, males may preferentially invest in SFPs that neutralise or buffer potential female immune responses against their sperm, or that contribute to make the female reproductive tract a more hostile environment for future inseminations by rival males. Interestingly, immunity and proteolysis regulators were also overrepresented within this group, including those that specifically contribute to complement activation and peptidase inhibition. The reason for why males preferentially invest in such SFPs later on in the mating sequence may be that males prioritise fertilisation in their first mating with a female, and only when they have opportunities to remate do they progressively switch to a strategy to protect the sperm they have already inseminated. Additionally, there is some evidence that transparent fluid might impair sperm fertilising performance in fowl^45^. If this were the case, males would be selected to temporally differentiate inseminations of sperm from those preferentially investing in transparent fluid proteins.

Preferential investment is also supported by the identification of protein abundance increases specific to the mating with the sexually novel female, including a highly significant enrichment of sperm proteins involved in axoneme structure, cilium movement, sperm motility and associated energetic pathways. The precise functional implications of this observation await further investigation, although we have previously speculated that these classes of molecules might contribute to post-testicular sperm maturation^37^. However, it is noteworthy that abundance increases in these proteins were unique in their highly correlated patterns of up-regulation. This suggests that preferential investment of SFPs associated with sexual novelty may be governed by a regulatory mechanism conferring tight patterns of co-expression.

Male social status emerged as a key predictor of variation in SFPs levels, from the Principle Component Analysis (PC3). Our results suggest that when mating with the second, novel female, subdominant males preferentially invested in SF proteins associated with sperm motility (e.g. flagellar functions, microtubule motor and cilium movement), whereas dominant males preferentially invested in proteins associated with exosomes, which might play a role in post-testicular sperm maturation^40^. The adaptive significance of these status-specific responses warrants further investigation. It is possible that subdominant males might respond to the higher risk of sperm competition typically faced by their ejaculates, by boosting the fertilising efficiency of an insemination through SFP investment. Sperm motility is a key determinant of the outcome of sperm competition in this species^47^, and is particularly important for males that are unable to top up their spermatic representation in a female through frequent mating^48^, as is typically the case for subdominant males^36,37^. Some studies have indicated that subdominant male fowl might produce more motile sperm than dominant males^38,49^, and have suggested that SF characteristics might underpin these differences^35,36^. Consistent with this hypothesis is recent compelling experimental evidence that status-specific variation in Chinook salmon *Oncorhynchus tshawytscha* sperm motility is caused by SF changes^34^. Corroborating evidence for the role of SFPs in sperm motility is accumulating across different taxa^24,50^, although identifying the molecular basis of such changes has proved more elusive. The status-specific patterns observed in SF proteomes confirm this general hypothesis, and suggest specific pathways through which SFPs might differentially influence the fertilizing efficiency of an ejaculate.

The propensity of dominant male fowl to invest preferentially in exosome-specific SFPs when mating with the second female suggests an alternative response. For example, given the possible roles of these exosomes in post-testicular sperm maturation, it is possible that dominant males might be depleting extragonadal reserves of mature sperm at a faster rate than subdominant males, forcing dominants to allocate sperm to the second female, which may have had less time for post-testicular maturation. Preferential investment of some SFPs may reflect other functions, e.g. manipulation of female post-mating responses such as propensity to remate or oviposition rate, as have been shown in other species^24^. Male fowl are able to manipulate female propensity to remate with other males through stimuli associated with mounting a female^51^. However, this does not eliminate scope for further manipulation through SFPs. Finally, it is possible that patterns of SFP allocation might reflect potential responses to interactions between competing ejaculates within the female. A male that is likely to mate with a female after another male may engineer the proteomic profile of his insemination to better exploit the SF effects elicited by the previous rival insemination^26,52^. For example, when SFPs stimulate female oviposition rate, the second male to mate with a female may exploit the stimulation investment of the first male and instead invest preferentially in the competitive fertilising efficiency of his ejaculate^26^. A response strikingly consistent with this expectation has been experimentally demonstrated in *Drosophila melanogaster*, where males mating first tend to preferentially invest in ovulin, a SFP stimulating female fecundity, compared to males mating with already mated females^28^.

Proteomic information on SFP variation is beginning to shed new light on ejaculate expenditure strategies, providing a platform to study ejaculate economics in a much more nuanced way than previously possible. For example, Sloan *et al*.^30^ have recently demonstrated that male crickets *Teleogryllus oceanicus*, down-regulate the expression of SFP genes when exposed to experimental simulations of elevated sperm competition intensity^30^. This pattern is broadly consistent with a fundamental prediction of ejaculate economic theory^5^, which has received ambiguous empirical support when investigated in terms of sperm number^7^. In our study, we found no evidence that dominant and subdominant males differ in their sperm allocation response to female novelty, as one could expect given that the relative role of sperm competition and sperm depletion is likely to vary considerably for socially dominant and subdominant males. However, our study did reveal a strong status-specific response in the proteomic profile of the SF produced over the sequence of ejaculates to the progressively sexually familiar first female and the second, sexually novel female. To conclude, global proteomic SF analysis reveals a refined molecular landscape of status-specific patterns of ejaculate expenditure that would be overlooked by traditional measures of sperm numbers, providing the foundation for a richer, more nuanced understanding of ejaculate expenditure behaviour, as well as the adaptive and functional significance of individual SFP variation. Future work should seek to support these qualitative patterns of SFP variation with more quantitative approaches based on larger samples, as well as investigate the adaptive significance of these responses by establishing the functional roles of individual SFPs in sperm competition and post-mating female responses.

## Materials and Methods

### Experimental design

We studied a captive population of red junglefowl at the John Krebs Field Station of the University of Oxford at Wytham (Oxfordshire), during the breeding season (May-August) 2014. We selected 26 males in order to create 13 social pairs housed in individual pens of 324 × 606 cm. The males used ranged from approximately 1 to 5 years old and were all in reproductive condition. We monitored their social interactions over two consecutive days between 16:00–19:00 h, starting one hour after the birds were released in the new pen, to allow the males to acclimatize to the new social and physical environment of the new pen. Social hierarchies between pair members were determined based on the outcome of competitive interactions using an established protocol^39,49^. The subdominant member of a pair was the male, which most frequently avoided (defined as a movement of at least one body length away from the approaching male) and was most frequently chased away or attacked by the other male (i.e. the dominant). In the two male pairs where the social status was not clear after the first two days of observations, we monitored their social interactions on the next day in 20-minutes watches between 14:00–16:00 h, and, as the hierarchy was still unclear, we repeated observations the following day while introducing a small amount of valuable resources in the pen (lettuce and egg yolk), in order to induce competition between the birds, after which the social status was always clear.

The goal of the experimental trials was to screen male propensity to invest in a sexually novel female under experimentally controlled conditions, across a set of dominant and subdominant males. The sample size of the experiment was therefore dictated by the balance between the need to obtain sufficient behavioural and proteomic detail for individual males with the need to characterise overall patterns across individuals. For each trial, a male pair was moved to an experimental pen, where each of the two males was kept in one half of the pen, physically separated from the other by a mesh partition, which kept them in visual and auditory contact. We allowed the males 30 minutes to acclimatize to the new pen before starting the ejaculate allocation trials. We conducted the trials between 14:00–20:00 h, following an established protocol^6,38,51–53^. A female was held for one minute facing the focal male in order to enable the male to inspect her. The female was then turned around and held in a soliciting position, placed on a plastic substrate and with a harness covering her cloaca to facilitate ejaculate collection without contact with the female reproductive tract. In order to prevent the male from being inhibited by the experimenter, one of us (AAF) sat behind a black plastic curtain with a square hole in its central bottom part through which the female was presented to the male. If a male did not copulate with a female in the first 10 minutes, the female was replaced and the procedure repeated. In total, each male was given the opportunity to copulate with up to 5 females, with a maximum of two females presented on any given day. The purpose of this first part of the experiment was to habituate males to copulate with a female under experimental conditions. When a male copulated with a female, we considered this female his first female and we started the experimental trial. Males required an average exposure to 2.05 ± 0.27 (mean ± SE) females to start mating with the first female. Two dominant and five subdominant males did not copulate with any of the 5 females to which they were presented during the habituation phase and, thus, were not included in the trials. The remaining 19 males ranged in age as follows: one 5 year old, seven 4-year old, four 3-year old, six 2-year old, and one 1-year old.

Following the first copulation, the male was given 10 more minutes to copulate a second time with the first female. If the male copulated a second time, he was given another 10 minutes to copulate a third time and so on for a total of up to 5 consecutive copulations with the first female. At the end of this session, the first female was immediately replaced by a new female, which was considered the second (sexually novel) female of the trial. Similarly, if following previous copulation(s) with the first female, the male failed to copulate with this female again, or copulated three consecutive times without delivering semen, the first female was replaced by the second female. Again, the second female was presented for one minute facing the male, and then turned around and held in soliciting position, placed on a plastic substrate and with a harness covering her cloaca. The male was given up to 10 minutes to copulate once with the second female, after which the trial was terminated. The focal males had not mated with the females to which they were exposed in the experiment for at least a month prior the trial. This design therefore means that the first female becomes sexually familiar to the male following the first mating, while the second female is novel to the male at the time of the first mating with this female. We repeated this protocol with both members of each male pair (Supplementary Fig. S7). Because males are known to bias ejaculate expenditure in response to female comb size^6,54^, we reduced variation in female comb size by using females with similar comb size within the same trial, so that there was no significant difference in comb size between the first and second female used during a trial (one-way ANOVA, F_1,24_ = 0.014, p = 0.906, Supplementary Fig. S8).

Immediately after each copulation, we collected the ejaculate and measured their volume using a Gilson pipette. Subsequently, 2.5 µl of semen were diluted into 197.5 µl of Phosphate Buffered Saline (PBS) and 70 µl of this mixture transferred to a spectrophotometer set at 595 nm to measure sperm concentration through the light absorbance. Sperm numbers were calculated from the ejaculate light absorbance based on an established standard curve. We then separated the sperm from the seminal fluid by centrifugation at 3,500 g for 1 minute at 4 °C, isolated the seminal fluid from the top of the supernatant, and froze it at −20 °C for protein quantification following an established protocol^40^.

All sampling of birds was conducted in accordance with approved experimental protocols under Home Office licence (PPL 30/2931).

### Seminal fluid protein quantification and fractionation

We selected a subset of trials (N males = 7, 4 dominant and 3 subdominant), in which males produced ejaculates with both first and second females. We first quantified the protein content of the seminal fluid of each sample (33 samples overall). Solubilization buffer (6 M Urea, 2 M Thiourea, 4% Chaps, 5 mM Mg Acetate, 10 mM Tris pH 8.5) was added to each seminal fluid sample and protein concentrations were quantified using the Quick Start Bradford Assay from Bio-Rad and a spectraMax M2 microplate reader. Bovine Serum Albumin (BSA) was used as a standard (scale for protein estimation from 0–1 mg/ml) and samples were sonicated using a bioruptor to disrupt residual protein interactions. To account for variation between males, protein was pooled from two dominant (B8 and J7) and two subdominant (B17 and G1) males, which produced samples for matings 1, 2 and 3 with the first female, plus the mating with the new, second female. 12.5 µg of protein was used per male for each of the four time points 25ug of total protein from each of the 8 samples was separated for the maximum duration possible (without running small proteins off the gel) on 4–12% Nupage Bis-Tris gels running on an XCell SureLock Mini-Cell PowerEase 200 system (Life-Technologies). Gels were then fixed in 45% methanol and 1.0% acetic acid for 1 hour and immediately stained with Coomassie (0.1%w/v Coomassie, 34% methanol, 17%w/v ammonium sulfate, and 0.5% acetic acid). Gels were transferred to a gel slicer where each lane was cut vertically and then horizontally into 5 slices, resulting in total of 40 samples for tandem mass spectrometry (MS/MS) analysis. Gel slices were subjected to proteolytic digestion using an Automated Preparation Station (Perkin Elmer). Proteins were first reduced and alkylated and then successively treated with dithiothreitol and iodoacetamide followed by digestion with trypsin (500 ng/µl, Promega) at 37 C for 16 h.

### Tandem mass spectrometry analysis

MS/MS experiments were performed using a Dionex Ultimate 3000 RSLC nanoUPLC (Thermo Fisher Scientific Inc, Waltham, MA, USA) system and an Orbitrap Lumos mass spectrometer (Thermo Fisher Scientific Inc, Waltham, MA, USA). Peptides were loaded onto a pre-column (Thermo Scientific PepMap 100 C18, 5 μm particle size, 100 A pore size, 300 μm i.d. x 5 mm length) from the Ultimate 3000 auto-sampler with 0.1% formic acid for 3 minutes at a flow rate of 10 μL/min. After this period, the column valve was switched to allow elution of peptides from the pre-column onto the analytical column. Separation of peptides were separated by C18 reverse-phase chromatography at a flow rate of 300 nL/min and a Thermo Scientific reverse-phase nano Easy-spray column (Thermo Scientific PepMap C18, 2μm particle size, 100 A pore size, 75μm i.d. x 50 cm length) at a flow rate of 300 nL/min). Peptides were loaded onto a pre-column (Thermo Scientific PepMap 100 C18, 5um particle size, 100 A pore size, 300μm i.d. x 5 mm length) from the Ultimate 3000 autosampler with 0.1% formic acid for 3 minutes at a flow rate of 10 μL/min. After this period, the column valve was switched to allow elution of peptides from the pre-column onto the analytical column. Solvent A was water + 0.1% formic acid and solvent B was 80% acetonitrile, 20% water + 0.1% formic acid. The linear gradient employed was 2–40% B in 93 minutes. (Total LC run time was 120 mins including high organic wash step and column re-equilibration). The eluted peptides from C18 column LC eluant were sprayed into the mass spectrometer by means of an Easy-Spray source (Thermo Fisher Scientific Inc.). All *m/z* values of eluting peptide ions were measured in an Orbitrap mass analyzer, set at a resolution of 120,000 and were scanned between *m/z* 375–1500 Da. Data dependent MS/MS scans (3 second cycle time) were employed to automatically isolate and fragment precursor ions and generate fragment ions by higher energy collisional-induced dissociation (HCD) (Normalised Collision Energy (NCE): 35%) in the ion routing multipole. The resolution of the Orbitrap was set to 15000 for the measurement of fragment ions. Singly charged ions, ions with greater than seven charges and ions with unassigned charge states were excluded from being selected for MS/MS and a dynamic exclusion window of 70 seconds was employed. Post-run, the data was processed and peak-lists generated using Protein Discoverer (version 1.3, ThermoFisher). The mass spectrometry proteomics data have been deposited to the ProteomeXchange Consortium http://proteomecentral.proteomexchange.org) via the PRIDE partner repository with the dataset identifier PXD010214.

### Peptide identification and protein annotation

Raw data from each MS/MS run was analyzed by X!Tandem^55^ and Comet^56^ against the complete *Gallus gallus* refseq protein database (June 2013 release, 32134 proteins), supplemented with 4 characterized, but yet to be annotated, immunoglobulin sequences. A fragment ion mass tolerance of 0.40 Da and a parent ion tolerance of 10.0 PPM were used. Iodoacetamide derivative of cysteine was specified as a fixed modification, whereas oxidation of methionine was specified as a variable modification. Peptides were allowed up to two missed trypsin cleavage sites. All downstream analyses were conducted using the Trans-Proteomic Pipeline - TPP v4.7 POLAR VORTEX rev 1^57^. False Discovery Rates (FDRs) were estimated with a randomized decoy database using PeptideProphet, employing accurate mass binning model and the nonparametric negative distribution model. X!Tandem and Comet PeptideProphet results were combined using iProphet, to provide more accurate and conservative identification probabilities. Peptide identifications were accepted if they could be established at greater than 95.0% iProphet probability and protein assignations were accepted if they could be established at greater than 99.0% probability. Proteins that contained identical peptides and could not be differentiated based on MS/MS analysis alone were grouped to satisfy the principles of parsimony.

### Protein abundance estimates

Protein quantitation was conducted using the APEX Quantitative Proteomics Tool^58^. Fifty SFPs previously identified using the same MS/MS methodology were utilized for the training dataset based on having the highest numbers of spectral counts per protein and the highest protein identification probabilities^40^. The 35 physicochemical properties available in the APEX tool were used for prediction of peptide detection/non-detection in the construction of a training dataset file. Protein probabilities (O_i_) were computed using the Random Forest classifier algorithm trained with the data set generated in the previous step and used to normalize APEX abundance estimates. APEX compositional protein abundances were calculated using the protXML file generated by the ProteinProphet. This analysis was conducted separately for all dominant and subdominant samples (n = 8) and combined subdominant and dominant samples for each successive mating (n = 4).

### Statistical analysis

#### Patterns of ejaculate expenditure

We first investigated the probability that males delivered an ejaculate over successive mating opportunities (i.e. female exposure) with a generalised linear mixed-effects model (GLMM), with a binomial error structure. Mating opportunities that resulted in mating and semen transfer were coded as 1 and those that did not were scored as 0. Explanatory variables included cumulative exposure order (continuous), female novelty (2-level factor) and their two-way interaction, and male identity as a random effect. Cumulative exposure measures the cumulative order of mating opportunities to which a male was exposed within a trial, across the first and second female. We expect ejaculate expenditure to decline with cumulative exposure due to depletion of the extragonadal sperm reserves of a male. Female novelty distinguishes between male responses to the first and second female. An effect of female novelty or an interaction between female novelty and cumulative exposure would enable us to identify male responses consistent with a preferential investment in female sexual novelty. We identified the most parsimonious models and the best minimal model using an Akaike Information Criterion (AIC) approach, corrected (AICc) because of data restrictions i.e. n/k <40.

We then focused on the subset of those mating opportunities in which semen was transferred and tested patterns of variation in sperm number, volume of seminal fluid and amount of protein (µg/µl) allocated to the seminal fluid of successive ejaculates produced over the mating sequence. To do this, we first conducted a linear mixed-effects model (LMM) with either, sperm numbers (log-transformed), seminal fluid volume (log-transformed) or protein concentration as the response variable, and utilised an AICc approach. We selected our top model set as those models within 2 AICc of the model with the lowest AICc. To estimate the effect sizes of parameters we used a model averaging approach where all variables were standardised to have a mean of zero and a standard deviation of 0.5 to facilitate interpretations of fixed effects^59,60^.

Testing the effect of female novelty is however complicated by the fact that males could only produce up to one ejaculate with the second female. We therefore corroborated this analysis with a complementary approach, which compared the number of sperm that a male delivered in his copulation with the second female with the number of sperm that he was predicted to deliver on the same copulation based on his pattern of decline of sperm numbers over previous copulations with the first female. Predictions for each male were made using a linear model of sperm number log + 1 transformed on cumulative exposure, including all exposures whether the male produced an ejaculate or not (in which case the male was assigned a zero). Predicted values were then back-transformed to sperm numbers and compared with observed sperm numbers with the second females using the paired Wilcoxon signed rank test. In cases where models predicted sperm numbers lower than zero, predictions were manually set to zero. We repeated this procedure for log + 1 transformed seminal fluid volume and protein concentration. All analyses were conducted using R packages “lme4” and “MuMin”^61–63^.

#### Hierarchical clustering, fuzzy clustering and Gene Ontology analyses

Heirarchical clustering was used to analyze APEX-normalized abundance estimates as implemented by the heatmap.2 from the “gplots” R package using default parameters. It is noteworthy that the distribution of protein abundance was comparable between samples (Supplementary Fig. S9). We first analyzed the inclusive set of 805 proteins identified in all four successive matings when dominant and subdominant proteomic data were combined and then the 607 proteins that were identified independently in samples from both dominant and subdominant males in all successive matings. SF samples and proteins were organized by dendrogram order, with the distance matrix computed by the dist R function using the euclidean method and the hclust R function using the complete linkage agglomeration method. Significance of clustering by approximately unbiased p-value^60^ and bootstrap probability was assessed using the “pvclust” R package using 10000 bootstrap replications^64^. Fuzzy clustering was performed on the log transformed APEX-normalized abundance estimates of the 805 proteins present in all matings, using the “Mfuzz” R package^65^. The optimality function was used to establish the most representative proximity of cluster components and cluster number was selected based on estimates from the minimum centroid distance and partial coefficient functions. Cluster membership was established based on the maximal membership values. Cluster composition was analyzed using Gene Ontology information from the DAVID Bioinformatics Resource (6.8)^66^, including statistical analyses of functional enrichment using the *G. gallus* genome as the background dataset for comparisons. The Piano software package was used to assess GO enrichment amongst proteins of differentiation abundance between dominant and subdominant samples using Z-score to assess levels of significance^67^.

#### Principal component analyses (PCA)

PCA was used to summarize variation using abundance estimates for the 607 proteins identified independently in samples from both dominant and subdominant males in all successive matings. Variation captured by each PC was determined using the “FactoMineR” R package^68^. We note that PC1 captured the majority of variation and this variation was highly correlated with protein abundance in all samples (average R-square = 0.896 (p < 0.0001 in all cases). We therefore focused our analyses on PC2 and PC3, which captured axes of variation between samples.

### Ethical approval statement

The work was conducted in accordance with protocols approved by the Animal Welfare Ethical Review Board (AWERB) of the Department of Zoology of the University of Oxford and under UK Home Office licence (PPL 30/2931).

## Supplementary information

Supplementary Information

Supplementary dataset 1

Supplementary dataset 2

## Data Availability

Data are available as Supporting Information.
