# Supplementary material for: Female novelty and male status dynamically modulate ejaculate expenditure and seminal fluid proteome over successive matings in red junglefowl
Source: Sci Rep. 2019 Apr 10;9:5852. doi: 10.1038/s41598-019-41336-5 (PMC6458113; doi:10.1038/s41598-019-41336-5)
Supplement: Supplementary file 1 — Supplementary Information [file 41598_2019_41336_MOESM1_ESM.pdf]

Supplementary Information

Female novelty and male status dynamically modulate ejaculate expenditure and seminal fluid proteome over successive matings in red junglefowl

Aitor Alvarez-Fernandez[1†], Kirill Borziak[2 †], Grant C. McDonald[1], Steve Dorus[2*], Tommaso Pizzari[1*]

[1]Edward Grey Institute, Department of Zoology, University of Oxford, UK

[2]Center for Reproductive Evolution, Syracuse University, 107 College Place, Syracuse, NY 13244, USA

[†] joint first authors

*corresponding authors: SD: sdorus@syr.edu; TP: tommaso.pizzari@zoo.ox.ac.uk

Ejaculate expenditure model averaging

Table S1. Effect of cumulative exposure (CE) and female order (F) on probability of ejaculation. Estimates are generated from model averaging over models with ΔAIC_C <2. Estimates are calculated using the zero method, where the estimates and standard errors (SE) are replaced with a zero in models where the parameter is absent. All models have the random effect for male identity. Models used a binomial error structure and effect sizes have been standardised to a mean of 0 and standard deviation of 0.5.

Parameter	Estimate	Adjusted SE	Confidence interval	Relative importance
Intercept	0.30	0.54	(-0.76, 1.36)	-
CE	-3.05	1.26	(-5.52, -0.57)	1.00
F	0.73	1.44	(-2.09, 3.56)	1.00
F × CE	2.25	2.17	(-1.99, 6.50)	0.67

Table S2. Effect of cumulative exposure (CE) and female order (F) on sperm numbers. Estimates are generated from model averaging over models with ΔAIC_C <2. Estimates are calculated using the zero method, where the estimates and standard errors (SE) are replaced with a zero in models where the parameter is absent. All models have the random effect for male identity. Response variable was log transformed and effect sizes have been standardised to a mean of 0 and standard deviation of 0.5.

Parameter	Estimate	Adjusted SE	Confidence interval	Relative importance
Intercept	18.55	0.15	(18.26, 18.85)	-
CE	-1.68	0.43	(-2.52, -0.84)	1.00
F	0.48	0.58	(-0.66, 1.61)	0.55

Table S3. Effect of cumulative exposure (CE) and female order (F) on seminal fluid volume. Estimates are generated from model averaging over models with ΔAIC_C <2. Estimates are calculated using the zero method, where the estimates and standard errors (SE) are replaced with a zero in models where the parameter is absent. All models have the random effect for male identity. Response variable was log transformed and effect sizes have been standardised to a mean of 0 and standard deviation of 0.5.

Parameter	Estimate	Adjusted SE	Confidence interval	Relative importance
Intercept	3.74	0.24	(3.28, 4.20)	-
CE	-0.75	0.37	(-1.47, -0.03)	1.00
F	0.16	0.53	(-0.88, 1.20)	0.56
F × CE	0.29	0.67	(-1.03, 1.61)	0.24

Table S4. Effect of cumulative exposure (CE) and female order (F) on protein concentration. Estimates are generated from model averaging over models with ΔAIC_C <2. Estimates are calculated using the zero method, where the estimates and standard errors (SE) are replaced with a zero in models where the parameter is absent. All models have the random effect for male identity. Effect sizes have been standardised to a mean of 0 and standard deviation of 0.5.

Parameter	Estimate	Adjusted SE	Confidence interval	Relative importance
Intercept	2.44	0.25	(1.96, 2.92)	-
CE	-0.55	0.25	(-1.04, -0.05)	1.00
F	0.10	0.25	(-0.38, 0.59)	0.30

Table S5. Means ± standard errors of the difference between observed values minus predicted values for absolute sperm numbers, seminal fluid volume and protein concentration for dominant and subdominant males. The difference is calculated by subtracting the predicted value from observed value. Positive values represent an average higher value for observed versus predicted values. Sample sizes (n) provided in brackets. Mann-Whitney U for Sperm numbers by social status: W = 41, p-value = 0.8323; Mann-Whitney U for Seminal fluid by social status: W = 34, p-value = 0.7121.

Variable	Status	
	Dominant	Subdominant
Sperm numbers	108,970,873 ± 76,466,439 (n = 11)	33,049,623 ± 20,395,745 (n = 8)
Seminal fluid volume	40.003 ± 25.317 (n =11)	28.099 ± 16.813 (n =7)
Protein concentration	0.384 ± 0.448 (n = 4)	0.606 ± 0.567 (n = 3)

Table S6. Means ± standard errors of the difference between observed values minus predicted values for standardised sperm numbers, SF volume and protein concentration for dominant and subdominant males. Values are standardised within males to the largest value observed in exposures with the first female (i.e. an observed value with the second female of 2 would thus mean a male delivered an ejaculate with a value twice the size of the largest value with the first female). The difference is then calculated by subtracting the predicted value from the observed. Positive values represent an average higher value for observed versus predicted values. Sample sizes (n) provided in brackets. Mann-Whitney U for Sperm numbers by social status: W = 34, p-value = 0.421; Mann-Whitney U for Seminal fluid by social status: W = 36, p-value = 0.854.

Variable	Status	
	Dominant	Subdominant
Standardised sperm numbers	0.069 ± 0.463 (n = 11)	0.028 ± 0.052 (n = 8)
Standardised SF volume	1.370 ± 1.313 (n =11)	0.190 ± 0.135 (n =7)
Standardised SF protein concentration	0.154 ± 0.183 (n = 4)	0.244 ± 0.232 (n = 3)

Table S7. Significant principal components, sample loadings and associated p-values.

	PC1	PC1	PC2	PC2	PC3	PC3	PC4	PC4	PC5	PC5
sample	coord.	p.value	coord.	p.value	coord.	p.value	coord.	p.value	coord.	p.value
dom.1	0.93	0.00	-0.26	0.00	0.16	0.00	-0.06	0.16	0.00	0.92
dom.2	0.95	0.00	-0.08	0.04	0.13	0.00	-0.04	0.28	0.08	0.05
dom.3	0.95	0.00	-0.05	0.23	0.07	0.07	-0.12	0.00	-0.21	0.00
dom.NF	0.90	0.00	0.33	0.00	0.17	0.00	0.24	0.00	-0.02	0.71
sub.1	0.92	0.00	-0.32	0.00	-0.08	0.06	0.09	0.02	0.07	0.08
sub.2	0.95	0.00	-0.04	0.29	-0.17	0.00	0.04	0.34	0.12	0.00
sub.3	0.94	0.00	0.07	0.08	-0.24	0.00	0.05	0.25	-0.15	0.00
sub.NF	0.89	0.00	0.39	0.00	-0.04	0.36	-0.20	0.00	0.11	0.01

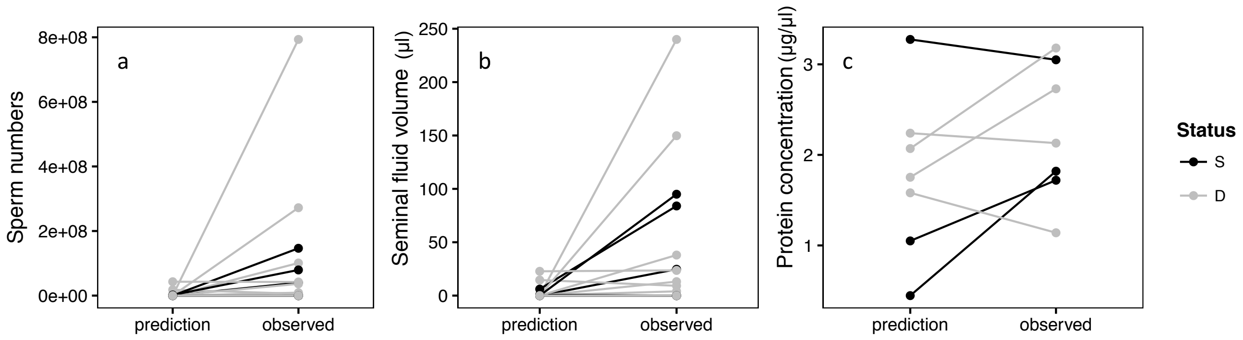

Fig. S1. Comparison of expenditure in last ejaculate with first female and ejaculate with second female for individual dominant (D) and subdominant (S) males, in terms of (a) sperm numbers, (b) seminal fluid volume, and (c) SF protein concentration.

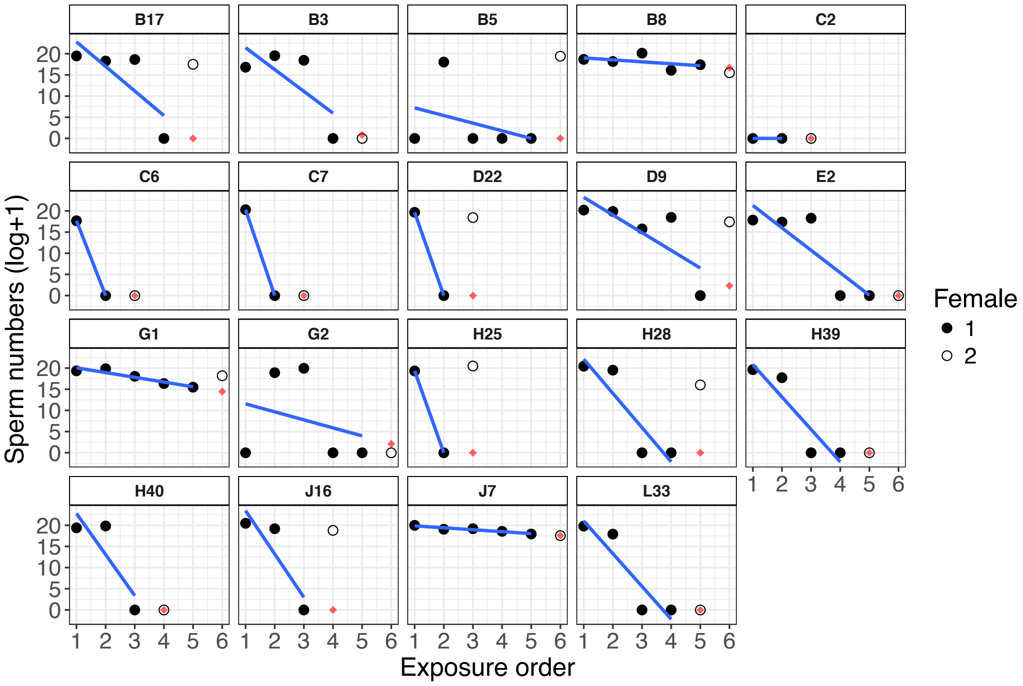

Fig. S2. Variation in sperm numbers delivered by individual males over the mating sequence of a trial. Filled circles represent exposures to the first female, open circles exposures to the second female. The red dot reflects the number of sperm that a male was predicted to invest in the second female based on pattern of sperm number decline in his copulations with the first female (i.e. based on models represented by blue lines).

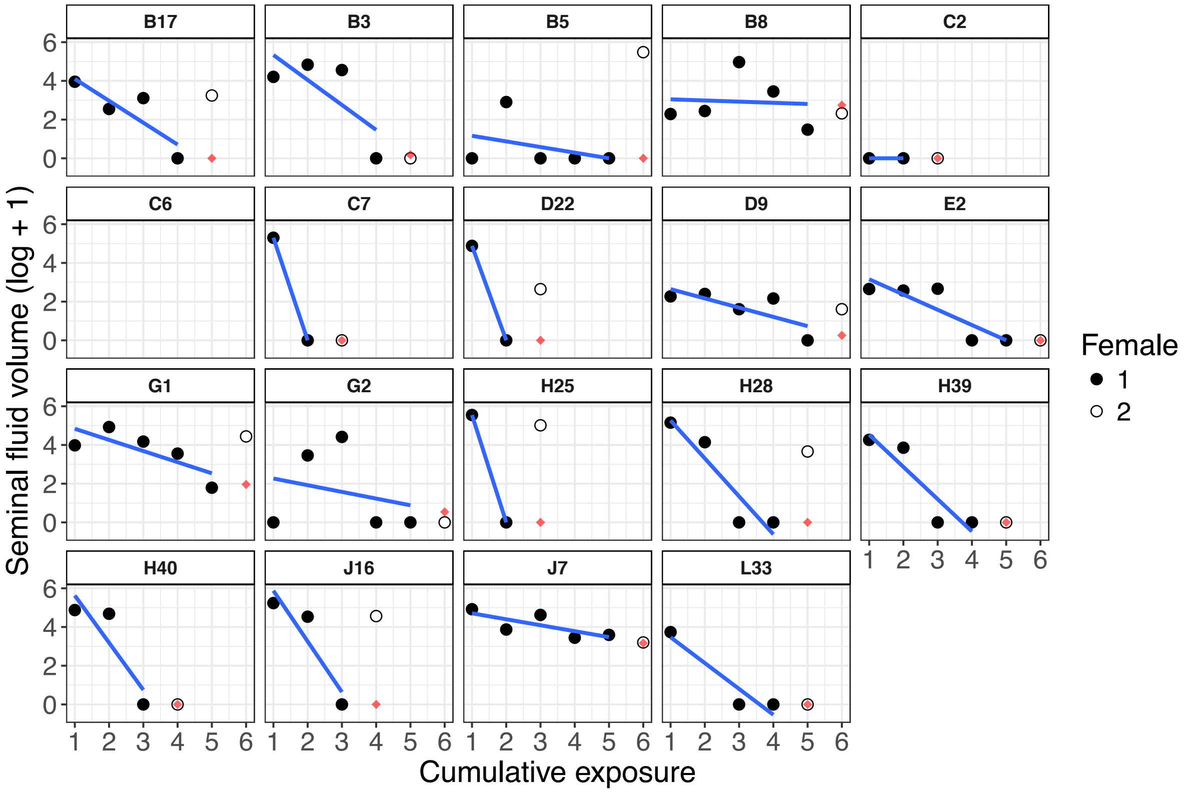

Fig. S3. Variation in volume of seminal fluid delivered by individual males over the mating sequence of a trial. Filled circles represent exposures to the first female, open circles exposures to the second female. The red dot reflects the seminal fluid volume that a male was predicted to invest in the second female based on pattern of seminal fluid volume decline in his copulations with the first female (i.e. based on models represented by blue lines).

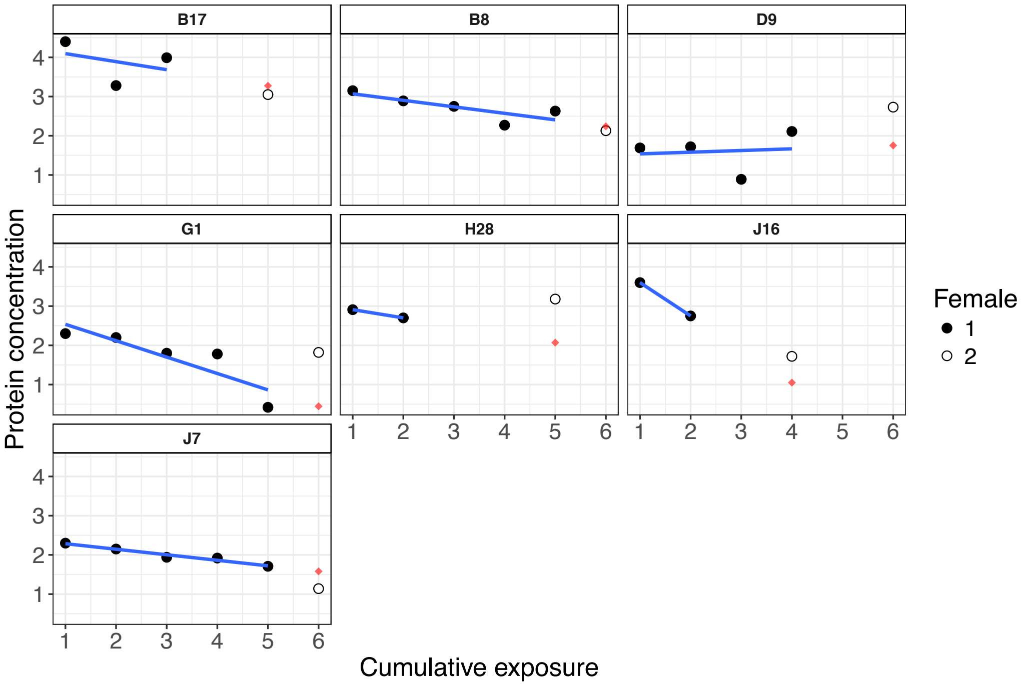

Fig. S4. Variation in seminal fluid protein concentration (μg/μl) delivered by individual males over the mating sequence of a trial. Filled circles represent exposures to the first female, open circles exposures to the second female. The red dot reflects the protein concentration that a male was predicted to invest in the second female based on pattern of protein concentration decline in his copulations with the first female (i.e. based on models represented by blue lines).

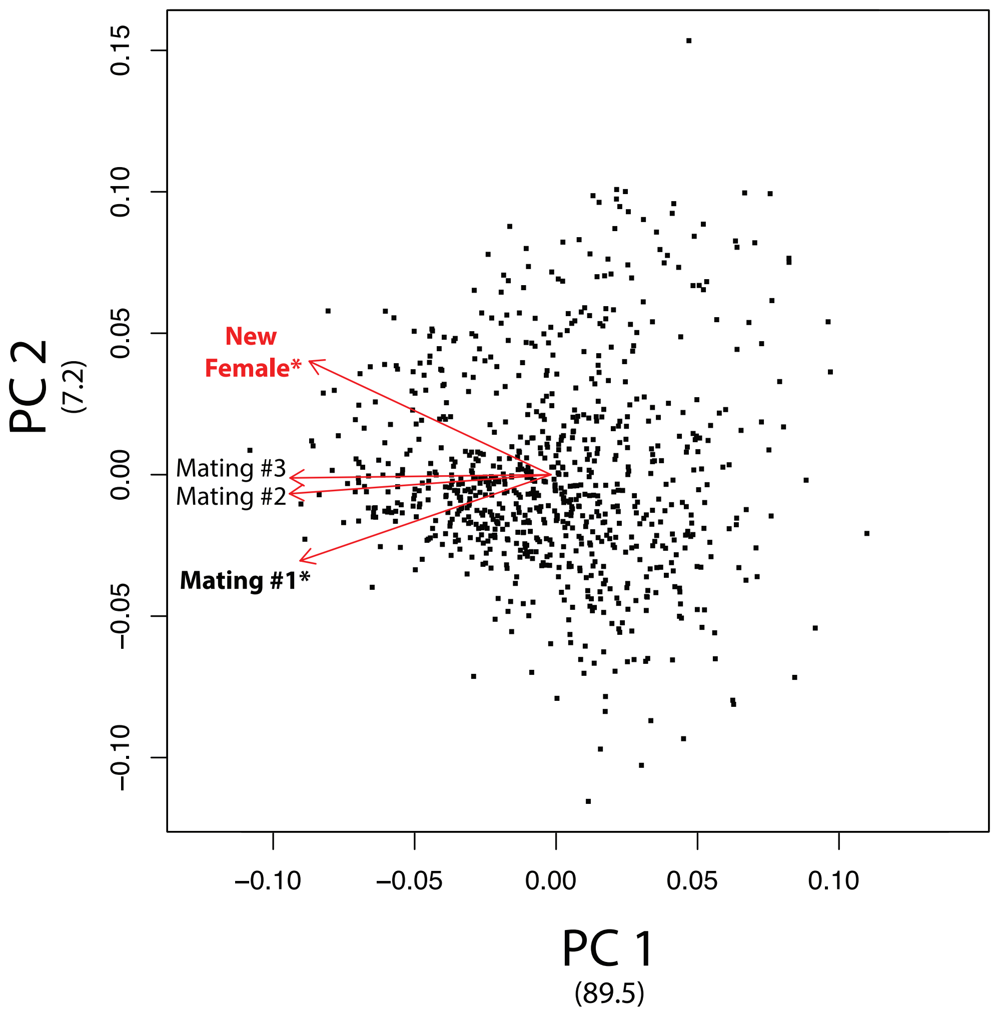

Fig. S5. Principal Component Analysis of SFP abundance across a mating sequence. Principal Component 1 (PC1) captured variance in the absolute abundance of SFPs (see Methods). PC2 loadings for the first mating were significantly negatively correlated with SFP abundance and PC2 loadings for the novel female mating were significantly positively correlated with SFP abundance. Thus, PC2 captured variance that distinguished between the first mating and the mating with the novel female. SFP composition was highly correlated between the second and third mating.

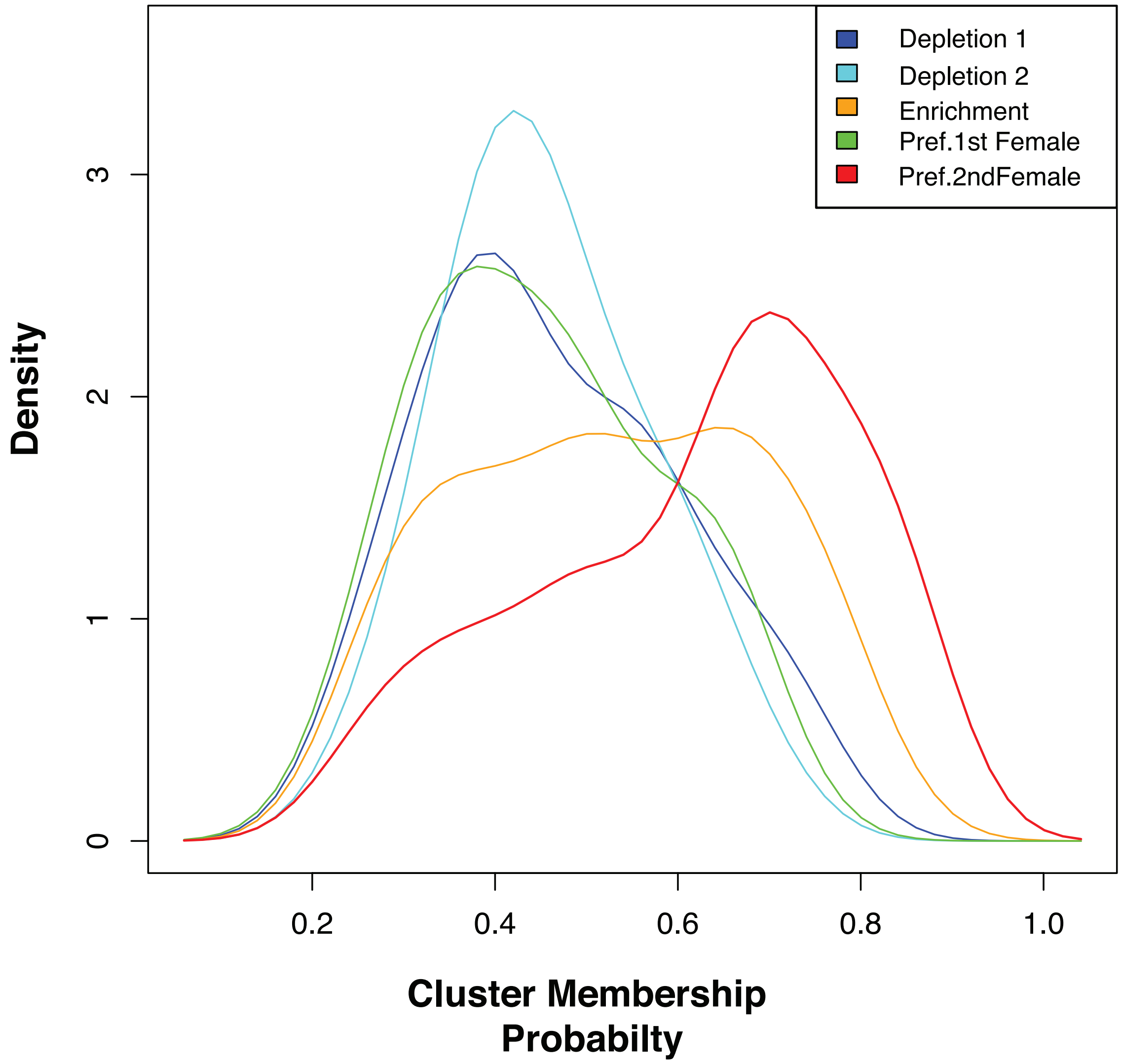

Fig. S6. Density distribution of membership probability for each cluster of proteins. Cluster membership was significantly greater for the "enrichment" and "preferential investment in 2nd female" clusters than each of the other clusters. This observation was consistent between cluster analyses using either four or five total clusters.

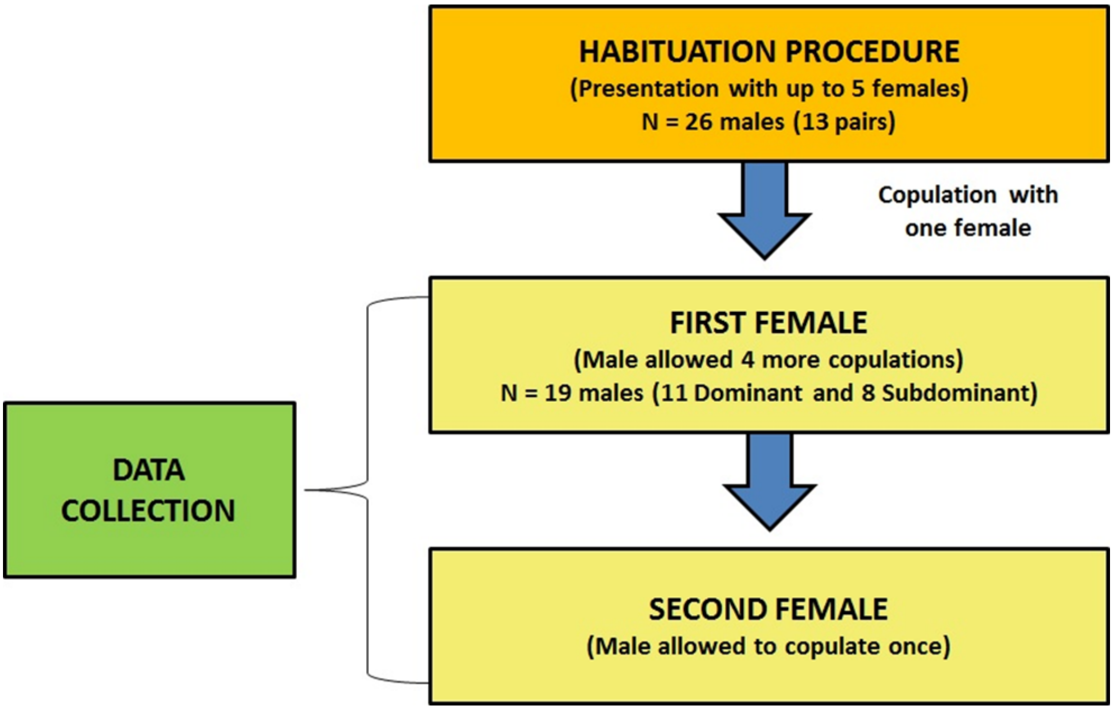

Figure S7. Experimental protocol outline.

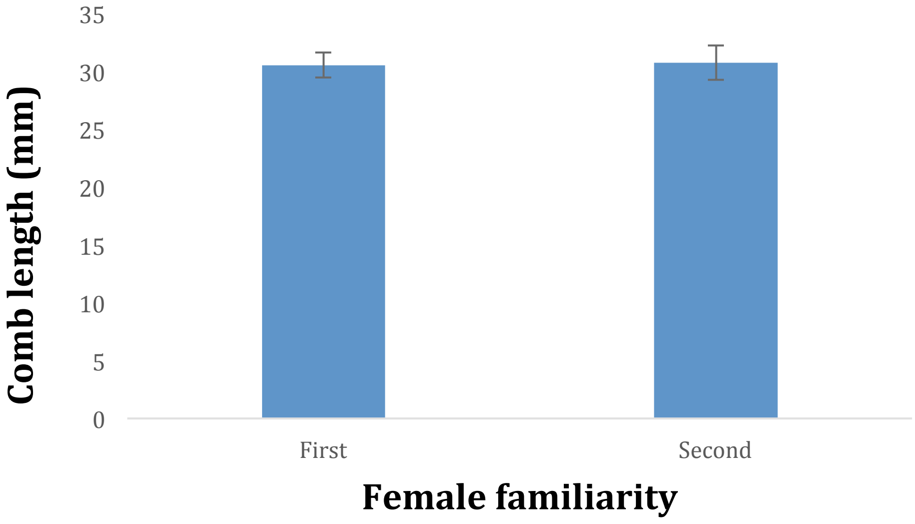

Figure S8. Comb size (mm) of first (i.e. progressively sexually familiar) and second (i.e. novel) females used during a trial.

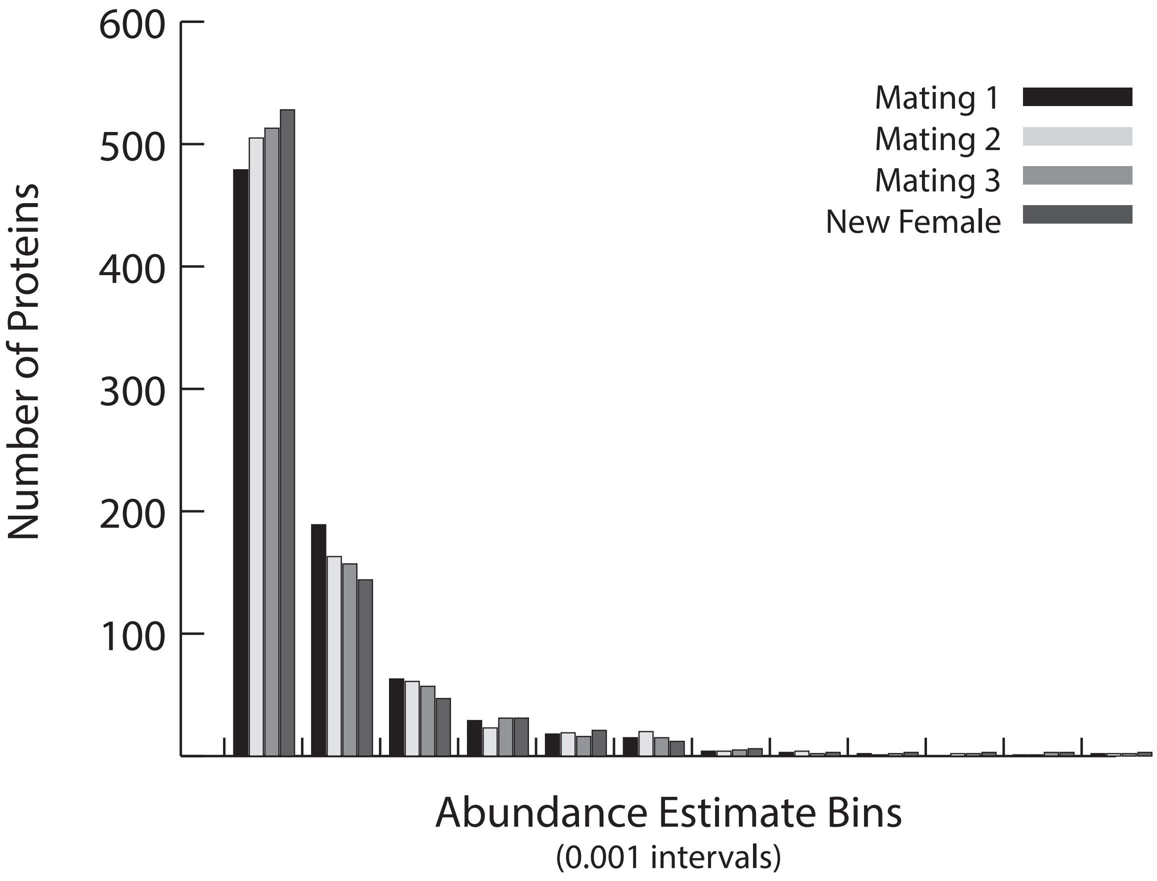

Figure S9. Distribution of APEX-normalized protein abundance estimates.
